# The impacts of the COVID-19 pandemic on the mental health and residency training of family medicine residents: findings from a nationwide cross-sectional survey in Turkey

**DOI:** 10.1186/s12875-021-01576-9

**Published:** 2021-11-15

**Authors:** Hüsna Çevik, Mehmet Ungan

**Affiliations:** 1 Çankaya District Health Directorate, Hacettepe Mah. Talatpaşa Blv. No: 44 Altındağ, Ankara, Turkey; 2 Family Medicine Department, Ankara University Medical School, Ankara, Turkey

**Keywords:** Burnout, COVID-19, Depression, Education, Family medicine, Graduate medical education, Pandemic, Primary care, Resident, Turkey, Work-related stress

## Abstract

**Background:**

The COVID-19 pandemic has had a negative impact on resident training in different branches and affected the physical and mental health of frontline residents adversely. This nationwide cross-sectional survey aimed to investigate the effects of the COVID-19 pandemic on family medicine residents in Turkey, including the levels of depression and burnout.

**Methods:**

An anonymous online survey was distributed to all family medicine residents via e-mail and a web link between 28.11.2020 and 12.12.2020. Information on sociodemographic data and the residency programme were evaluated, and factors associated with depression and burnout were examined using the Patient Health Questionnaire-9 (PHQ-9) and the Burnout Measure-Short Version (BMS) respectively.

**Results:**

Although the weekly average working hours of the 477 respondents increased significantly during the pandemic (*p* < 0.05), the average weekly working time in the Family Medicine (FM) outpatient clinic decreased. The greatest concern of 58.3% of the residents was fear of transmitting COVID-19 to their family members. 90.2% of the residents stated that training programmes were negatively or very negatively affected. According to PHQ-9 scores, 15.7% of residents had moderately severe, and 14.9% severe depression. The BMS scores of the residents demonstrated that 24.1% had a very severe burnout problem, and 23.3% should seek professional help as soon as possible. Being single, having no children, female gender, lack of personal protective equipments and increased contact time with COVID-19 patients were associated with higher scores in the depression and burnout scales (*p* < 0.05).

**Conclusions:**

The COVID-19 pandemic has had a negative impact on training programmes for FM residents, who are at the forefront of the pandemic in Turkey, and this situation is closely related to depression and burnout. Due to the unpredictability of the pandemic, long-term plans should be made for the training needs of residents in order to protect their physical and mental health.

**Supplementary Information:**

The online version contains supplementary material available at 10.1186/s12875-021-01576-9.

## Background

The World Health Organization (WHO) defined COVID-19 as a pandemic on March 11, 2020 consequent to the spread and severity of the virus [1]. Many hospitals, emergency rooms, almost all in-patient departments, and intensive care units were suddenly overwhelmed with suspected and confirmed COVID-19 patients, and functioned at full capacity [2]. As of 17 June 2021, more than 5 million people in Turkey have been infected with COVID-19 and the number of deaths has exceeded 49 thousand including a considerable number of healthcare professionals [3]. Since the beginning of the COVID-19 pandemic, measures have been taken to reduce the spread of the virus such as stopping reciprocal flights to countries with a high number of cases, closing kindergartens and schools, and implementation of legal arrangements and incentives to provide opportunities to work from home. Curfew for people aged above 65 and for young people aged under 20, temporary curfews, and a full lock-down on April 2021 due to the extreme increase in COVID-19 cases in Turkey have been implemented by the government. Pandemic hospitals were established in anticipation of increasing case numbers. By April 2020, contact tracing (filiation studies) teams were established for the purpose of finding cases and contact tracing in the field of pandemic control [2, 4, 5] More than 38 million doses of COVID-19 vaccines have been administered to date [3].

The pandemic related unpredictable circumstances forced healthcare systems to rapidly reorganize their network to optimize resources and reduce the spread of COVID-19 and within this framework, the COVID-19 pandemic resulted in the switch from person-centered care to community-centered care. Increased incidents of the COVID-19 infection forced healthcare professionals to put other preventive health services and management of non-communicable diseases (NCD) on the back burner, and has resulted in a decrease of person-centered care [6]. Supplies, medical devices, and drugs were produced, purchased, and procured at short notice within Turkey and all healthcare system employees were redeployed to manage COVID-19 patients. Overall, this trajectory has rapidly led to a substantial decrease in medical practice other than COVID-19, like NCD management, in Turkey [7] and consequently, may result in ongoing disruption threatening the continuum of residency training [8].

WHO has stated that countries should strengthen their primary care systems in the fight against the COVID-19 pandemic. In addition to pandemic action plans, such as determining potential cases as soon as possible, it is also expected that primary healthcare services should continue to be responsible for the well-being of the individual and society, by ensuring the provision of primary healthcare and preventive medicine practices, including vaccination and infant-child monitoring [9]. Family doctors, as a primary healthcare provider, have an important role during the pandemic, with comprehensive healthcare services independent of age and gender provided by accessible primary care clinics (PCC) [10]. FM residency training increases the quality of primary care services in line with WHO recommendations. Clinical training of residents to adopt FM core competencies and rotation training are required for a comprehensive and holistic approach. Studies conducted in FM clinics during the pandemic have shown that one of the main problems in resident training is the uncertainty about how their rotations will be affected by the pandemic in the long term [11].

It has been stated in literature that healthcare professionals require mental health support during the pandemic and that the implementation of relevant policies and interventions by managers is a vital need [12–14]. Mental health disorders including post-traumatic stress disorder, anxiety disorder, depression and burnout syndrome occur in physicians due to working conditions, increased stress, resource limitations, and uncertainty of physical safety [12]. It has been reported that the uncertainty resulting from the interruption of training programs and rotations, as well as the constant change of planned workplace rotations, due to the pandemic has also negatively affect the mental health of physicians. As such, an education programme should be intentionally prioritized [15]. Worldwide, face-to-face training programmes were translated to online formats and thus the continuation of the training programmes was ensured [16].

Residents have the most prolonged direct contact with suspected or confirmed cases of COVID-19, resulting in enhanced mental and physical stressors [17] and it is impossible to ignore the adverse impact the COVID-19 pandemic has had on residents, residency training and primary health care services. The aim of this nationwide survey was to investigate the effects of the COVID-19 pandemic on FM residents in Turkey, including the levels of depression and burnout in this period.

## Methods

### Participants and sampling

There were a total of 2765 FM residents in Turkey on 01/11/2020. All FM residents within the scope of the training programme were included in the study. As the present study is a descriptive, cross-sectional nationwide study, there was no need for sample size calculation.

The survey was uploaded and shared on the Google online survey platform and was delivered to FM residents through the Turkish Association of Family Physicians (TAHUD) e-mail group and WhatsApp groups of FM residents. The survey could be accessed through a web link given in the e-mail. The online questionnaire was sent to all FM residents who were continuing their education in Turkey between 28.11.2020 and 12.12.2020. A reminder e-mail was sent 1 week after the first posting, and data was collected anonymously over 2 weeks. The questionnaire was completed on a voluntary basis, incomplete answers to the questions was an exclusion criterion, and the results were based on completed survey responses.

### Study settings

There are two ways to serve as a family physician in Turkey. The first is that medical school graduates work in PCCs without any specialized training. The second way is to become an FMS after residency training.

Family Medicine Residency in Turkey is a 3-year postgraduate training programme, and it involves practicing in inpatient and outpatient settings. Eighteen months of the programme consists of several rotations: internal medicine, paediatrics, obstetrics&gynecology, psychiatry, emergency medicine, dermatology, pulmonary medicine, cardiology, and an elective rotation (general surgery or physical therapy and rehabilitation or neurology). Physicians who have graduated from the Faculty of Medicine are placed in the specialty of FM by successfully passing a National Central Examination for Specialization in Medicine. FM residency training is provided by the departments in the Universities and Training and Research Hospitals (TRH).

Contracted FM resident (CFMR) training is a form of FM residency supported by the government to increase the number of family medicine specialists rapidly in which general practitioners working in PCC become an FMS through completing rotation training over 6 years in a University Hospital or TRH.

### Instrument and measures

Participants were asked 23 close-ended questions regarding sociodemographic information (age, gender, marital status, having children); the details of the residency programme (the time spent in the practice of medicine and FM residency, place of work, type of residency, weekly hours of work in total and in the FM outpatient clinic, the duration and types of participation in the weekly education programmes, the changes made in the residency programme due to the pandemic, the effect of such on the residents, risk status [subjective evaluation of residents] of the FM Specialists (FMS) and residents during the pandemic); contact with COVID-19 patients (weekly total contact time, place of contact); the greatest concern during the pandemic, and protection from COVID-19 (training to deal with the pandemic and use of personal protective equipment [PPE], PPE availability, being infected by the COVID-19, treatment). For the questions of types of participation in weekly education programmes, the changes made in the residency programme due to the pandemic, and the effect of such on the residents had multiple choice options. Impact of the COVID-19 pandemic on the knowledge of some areas on the residents was measured by a 5-point Likert-scale (between 1 ‘very negatively affacted’ and 5 ‘very positively affected’). Two scales were used to assess the mental health of the participants; the Patient Health Questionnaire-9 (PHQ-9) [18] to determine the level of depression, and the Burnout Measure-Short Version (BMS) [19] to determine the burnout status (Table 1). This questionnaire was prepared with consideration to previous studies investigating the impact of the COVID-19 pandemic on resident training [8, 20, 21].

**Table 1:** The calculation of the PHQ-9 and BMS, and the scores of respondents

Score	Description	% (n) of residents
**Patient Health Questionnaire-9** (includes 9 questions each scored between 0 and 3)		
1–4	Minimal depression	9.6% (*n* = 46)
5–9	Mild depression	32.7% (*n* = 156),
10–14	Moderate depression	27.0% (*n* = 129),
15–19	Moderately severe depression	15.7% (*n* = 75),
20–27	Severe depression	14.9% (*n* = 71).
The average score of residents: 11.99 ± 6.47 (median:11)		
**Burnout Measure-Short Version** (calculated by totaling the score of 10 items [scored from “1 Never” to “7 Always”] and dividing by 10)		
≤2.4	A very low degree of burnout	11.9% (*n* = 57)
2.5–3.4	There are danger signals for burnout	14.9% (*n* = 71)
3.5–4.4	A state of burnout	25.8% (*n* = 123)
4.5–5.4	A very severe burnout problem	24.1% (*n* = 115)
≥5.5	Should seek professional help as soon as possible	23.3% (*n* = 111)
The average score of residents: 4.31 ± 1.42 (median:4.3)		

The validated Turkish version of PHQ-9 includes 9 questions and each question scored between 0 (not at all) and 3 (nearly every day) [18, 22]. Points are summed up for every question. Points between 1 and 4 are rated as minimal, 5–9 mild, 10–14 moderate, 15–19 moderately severe, and 20–27 severe depression according to the scoring system of the original questionnaire.

The validated Turkish version of 10-item BMS is a self-assessment scale [19, 23]. It consists of 10 questions relating to work-related situations, the professional environment and the ways in which these are experienced. A score between 1 (Never) and 7 (Always) is given for each situation. For the scale score calculation, the scores given to the 10 items are added together and divided by the number 10. The result is the occupational burnout score. This single total score is interpreted as the burnout level as follows; ≤2.4 points: a very low level of burnout, 2.5–3.4: there are danger signals for burnout, 3.5–4.4: a state of burnout, 4.5–5.4: a very serious burnout problem, and ≥ 5.5: professional help should be sought as soon as possible.

### Ethical considerations

Ankara University Faculty of Medicine Human Research Ethics Committee approval (27.11.2020/İ10–641-20) was obtained for this study. The study was performed in compliance with the principles of the Helsinki Declaration. The inclusion of participants was voluntary and informed consent was obtained from every participant. The confidentiality and anonymity of respondents were also guaranteed.

### Statistical analysis

The data obtained in the study were analyzed using IBM SPSS version 20 software (SPSS Inc., Chicago, IL). Descriptive statistics were reported as mean (±standard deviation) values for variables with normal distribution, as median (minimum-maximum) values for variables with non-normal distribution, and as number (n) and percentage (%) for nominal variables. In the comparison of two groups, the significance of the difference between the groups was investigated with the t-test or Mann-Whitney-U test as appropriate. When the number of groups was more than two, the significance of the difference between the groups was investigated using One Way Analysis of Variance (ANOVA) or Kruskal Wallis Test. Nominal variables were evaluated with the Pearson Chi-Square/Fisher’s Exact Test. The effects of the independent variables on the scores obtained from the PHQ-9 scale and BMS scale were examined with univariate and multivariate linear regression analysis. Independent variables that were found to be significant in univariate analyzes which influenced scale scores were included in the multivariate regression model. In multivariate regression analysis, the effects of the independent variables on scale scores were tested together. A value of *p* < 0.05 was considered statistically significant.

## Results

### Respondents’ sociodemographic and working characteristics

There were a total of 2765 FM residents in Turkey on 01/11/2020. The questionnaire was answered by 493 FM residents (Response rate: 17.25%). After the exclusion of 16 participants because of incomplete responses in the questionnaire, the study was conducted on a total of 477 residents (Table 2).

**Table 2:** The sociodemographic data and working conditions (during the pandemic) of FM residents responding to the questionnaire

**Gender**	
Male	37.3% (*n* = 178)
Female	61.2% (*n* = 292)
Not stated	1.5% (7)
**Age**	Median:28 (range, 24–54 years)
**Marital status**	
Married	59.1% (*n* = 282)
Single	39.2% (*n* = 187)
Divorced	1% (*n* = 5)
Widowed	0.6% (*n* = 3)
**Had children**	31% (*n* = 148)
**Duration of medical practice**	Median:5 (range, 0–30 years)
**Time spent in FM residency**	Median:12 (range, 0–75 months)
**Type of residency**	
Full-time FM residents	72.3% (*n* = 345)
Contracted FM residents (CFMR^a^)	27.7% (*n* = 132)
**Place of work**	
University Hospital	38.4% (*n* = 183)
Training and Research Hospital (TRH)	35.0% (*n* = 167)
Primary Care Clinic (PCC)	26.6% (*n* = 127)
**Greatest concerns during the COVID-19 pandemic**	
Clinical disability	7.8% (*n* = 37)
Falling behind in education	21.4% (*n* = 102)
Ethical issues	2.1% (*n* = 10)
The fear of catching COVID-19	10.5% (*n* = 50)
The fear of carrying COVID-19 to the family	58.3% (*n* = 278)
**Average weekly contact time with COVID-19 + patients (hours)**	
None	3.1% (*n* = 15)
< 8	17.6% (*n* = 84)
8–16	10.7% (*n* = 51)
16–24	15% (*n* = 76)
24–32	15.3% (*n* = 73)
32–40	15.9% (*n* = 76)
> 40	21.4% (*n* = 102
**Location of contact with patients with suspected or confirmed COVID-19**	
FM outpatient clinics	50.1% (*n* = 239)
COVID-19 outpatient clinic	40.9% (*n* = 195)
Emergency department	25.4% (*n* = 121)
COVID-19 inpatient treatment unit	46.1% (*n* = 220)
COVID-19 Intensive Care Unit (ICU)	7.8% (*n* = 37)
Contact tracing (filiation) studies^a^	23.7% (*n* = 113)
**Changes made to the residency programme due to the COVID-19 pandemic**	
No changes	9% (*n* = 43)
Residents were dismissed from some services	13.6% (*n* = 65)
Residents were working shifts	20.8% (*n* = 99)
Unable to obtain leave from work (although the Ministry of Health had officially stated that annual leave could be taken)	10.9% (*n* = 52)
Able to take leave after official notification	20.5% (*n* = 98)
Assigned to outpatient clinics and services other than FM	72.5% (*n* = 346)
Some changes had been made temporarily, but the residency programme had then returned to normal	5.9% (*n* = 28)
**PPE availability**	
All PPEs have been provided by the institution where they worked	31.0% (*n* = 95)
Some PPEs have been provided by the institution where they worked	57.2% (*n* = 273)
Residents provided their own PPEs	11.7% (*n* = 56)
**Being infected with the COVID-19**	19.9% (*n* = 95)
Continued working without treatment	4.2% (*n* = 4)
Stayed in quarantine without receiving treatment	9.5% (*n* = 9)
Treated at home	80.0% (*n* = 76)
Received inpatient treatment	6.3% (*n* = 6)

^a^Filiation is the contact tracing of confirmed COVID-19 cases

Although the weekly average working hours of the residents increased significantly during the COVID-19 pandemic (*p* < 0.05), the average weekly working time in the FM outpatient clinic decreased (Table 3).

**Table 3:** Respondent working hours and duration of participation in training programmes, and types of and changes to education programmes before and during the pandemic

**Average working time per week (hours)**	**Before the COVID-19 Pandemic [n (%)]**		**During the COVID-19 Pandemic [n (%)]**	
< 24	15 (3.1%)		15 (3.1%)	
24–40	184 (38.6%)		108 (22.6%)	
40–56	227 (47.6%)		226 (47.4%)	
56–72	30 (6.3%)		67 (14%)	
> 72	21 (4.4%)		61 (12.8%)	
**Average working time per week in the FM clinic (hours)**	**Before the COVID-19 Pandemic [n (%)]**		**During the COVID-19 Pandemic [n (%)]**	
< 8	92 (19.3)		164 (34.4%)	
8–16	58 (12.2%)		64 (13.4%)	
16–24	49 (10.3%)		37 (7.8%)	
24–32	63 (13.2%)		44 (9.2%)	
> 32	215 (45.1%)		168 (35.2%)	
**Duration of participation in training programmes per week (hours)**	**Before the COVID-19 Pandemic [n (%)]**		**During the COVID-19 Pandemic [n (%)]**	
< 1	56 (11.7%)		219 (45.9%)	
1–3	213 (44.7%)		161 (33.8%)	
3–5	173 (36.3%)		79 (16.6%)	
> 5	35 (7.3%)		18 (3.8%)	
**Education methods**	**Face to face before the COVID-19 pandemic [n (%)]**	**Online before the COVID-19 pandemic [n (%)]**	**Face to face during the COVID-19 pandemic [n (%)]**	**Online during the COVID-19 pandemic [n (%)]**
**Literature research**	286 (60%)	2 (0.4%)	33 (6.9%)	228 (47.8%)
**Seminars and/or lectures delivered by residents**	382 (80.1%)	0 (0%)	49 (10.3%)	289 (60.6%)
**Seminars and/or lectures delivered by faculty members at the institution**	361 (75.7%)	6 (1.3%)	41 (8.6%)	294 (61.6%)
**Seminars and/or lectures delivered by faculty members from another institution**	268 (56.2%)	47 (9.9%)	22 (4.6%)	284 (59.5%)

Contact with suspected or confirmed COVID-19 patients [24] in the FM outpatient clinics was reported by 96.2% (*n* = 127) of contracted FM residents (CFMR) who provided primary care in the field during periods other than their rotations, and by 32.5% (*n* = 112) of full-time FM residents. CFMRs stated that they were in contact with suspected or confirmed COVID-19 patients in FM outpatient clinic at a significantly high rate than full-time FM residents (*p* < 0.001). CFMRs were also significantly more likely to report that no change was made in the residency programme during the COVID-19 pandemic (*p* < 0.001) (Table 2).

19.9% (*n* = 95) of the residents who stated that they had had COVID-19 infection, 46.3% (*n* = 44) were in contact with COVID-19 positive patients for an average of 32 h or more weekly, 25.2% (*n* = 42) worked in a TRH, 20.8% (*n* = 38) worked in a University Hospital and 11.8% (*n* = 15) worked in a PCC. A significant difference was seen between the groups according to place of work (*p* = 0.017). Cross-tabulation analysis showed that the frequency of COVID-19 infection in doctors working in a TRH was significantly higher than that of residents working in a PCC (*p* < 0.05).

There was no significant difference between the time spent in residency and the average weekly contact time with COVID-19 positive patients (*p* = 0.81).

A significant difference was found between the residency status of the participants and the average weekly contact time with COVID-19 positive patients (*p* < 0.001), with 67.2% (*n* = 232) of full-time FM residents and 14.4% (*n* = 19) of CFMRs reported ≥24 h per week exposure. While 68.3% (*n* = 114) of residents working in TRHs and 65.6% (*n* = 120) of residents working in University Hospitals reported exposure of ≥24 h per week, the rate was only 14.4% (*n* = 19) in PCCs.

Contact with COVID-19 positive patients in the Intensive Care Unit (ICU) was reported by 16.2% (*n* = 27) of the residents working in a TRH, as opposed to 4.9% (*n* = 9) in University Hospitals, and 0.8% (*n* = 1) during the rotation of CFMRs working in PCCs. The frequency of contact with COVID-19 positive patients in ICU thus showed a significant difference according to institution (*p* < 0.001). In the cross-tabulation analysis it was found that residents working in a TRH have significantly more frequent COVID-19 positive patient contact in ICU compared to the residents working in a PCC (*p* < 0.05).

### Changes in FM residency education and training during the pandemic crisis

Online seminars given by external training staff increased significantly during the pandemic, compared to the pre-COVID-19 period (*p* < 0.001). Whilst only 16.8% (*n* = 58) of full-time FM residents attended training programmes for 3 h or more per week, 29.5% (*n* = 39) of contracted FM residents (CFMR) attended training programmes for 3 h or more (*p* < 0.01) (Table 3). 78.0% of the residents (*n* = 372) reported that the FMSs and/or faculty members they worked with took fewer risks compared to the residents themselves. Inequality of working pandemic-related tasks between residents and FMSs/faculty members was reported by 79.2% (*n* = 378) of the residents.

Specific training on how to deal with COVID-19 and use PPE was received by 37.9% (*n* = 181) of the residents before the first case was reported in Turkey, and by 74% (*n* = 353) following the first reported case, while 26% (*n* = 124) received no such training at any time. There was no significant correlation between the PPE training status of the residents and the frequency of having COVID-19 infection (*p* > 0.05).

### Impacts of the COVID-19 pandemic on psychological well-being of FM residents (Depression & Burnout)

More than 90% of residents reported that their mental health was negatively or very negatively affected (Table 4).

**Table 4:** Impact of the COVID-19 pandemic on education and health

Reported effect of COVID-19 pandemic	Very negatively affected [% (n)]	Negatively affected [% (n)]	Not affected [% (n)]	Positively affected [% (n)]	Very positively affected [% (n)]
Education programmes	43.2% (*n* = 206)	47.0% (*n* = 224)	6.7% (*n* = 32)	2.5% (*n* = 12)	0.6% (*n* = 3)
Educational experience in inpatient clinics	31.7% (*n* = 151)	44.0% (*n* = 210)	13.8% (*n* = 66)	10.5% (*n* = 50)	0% (*n* = 0)
Educational experience in outside rotations	42.6% (*n* = 203)	43.8% (*n* = 209)	13.2% (*n* = 63)	0% (*n* = 0)	0.4% (*n* = 2)
Physical health	28.5% (*n* = 136)	49.3% (*n* = 235)	21.8% (*n* = 104)	0.4% (*n* = 2)	0% (*n* = 0)
Mental health	52.0% (*n* = 248)	38.6% (*n* = 184)	9.2% (*n* = 44)	0% (*n* = 0)	0% (*n* = 0)

The results of the PHQ-9 and BMS questionnaires are shown in Table 1. The relationship between the questionnaire responses and the PHQ-9 and BMS scores were analyzed and significant results are shown in Table 5. The PHQ-9 and BMS scores showed statistically significant differences across marital status, gender, having children, type of residency, place of work, PPE availability and weekly contact time with confirmed COVID-19 positive patients. In cross-tabulated analyses to explore significant group differences, the PHQ-9 (*p* = 0.006) and BMS (*p* = 0.002) scores of married residents were determined to be significantly lower than those of single residents; the PHQ-9 (*p* < 0.001) and BMS (*p* < 0.001) scores of females were observed to be significantly higher than those of males; the PHQ-9 (*p* = 0.001) and BMS (*p* < 0.001) scores of residents working in a TRH were observed to be significantly higher than those of residents working in a PCC; the PHQ-9 (*p* = 0.005) and BMS (*p* = 0.012) scores of residents who provided their own PPE were observed to be significantly higher than those of residents who had their PPE provided by their institutions, and the PHQ-9 (*p* = 0.002) and BMS (*p* < 0.001) scores of doctors with < 8 h patient contact were determined to be significantly lower than those of the residents with > 40 h contact. In contrast, no statistically significant difference was found in the PHQ-9 and BMS scores respective to having or not having been infected with COVID-19 (Table 5).

**Table 5:** Analysis of sociodemographic and COVID-19 categories against PHQ-9 and BMS scores

**Marital status**	**Patient health questionnaire-9**	***p*** **value**	**Burnout Measure-Short Version**	***p*** **value**
Married	Median:10 (Min:0, Max:27)	0.006**	Median:4.2 (Min:1, Max:7)	0.02*
Single˟	Median:12 (Min:0, Max:27)		Median:4.5 (Min:1, Max:7)	
**Having a child**	**Patient health questionnaire-9**	***p*** **value**	**Burnout Measure-Short Version**	***p*** **value**
Yes	Median:9 (Min:0, Max:26)	0.001**	Median:4,05 (Min:1, Max:7)	< 0.001**
No	Median:12 (Min:0, Max:27)		Median:4,5 (Min:1, Max:7)	
**Average weekly contact time with COVID-19(+) patients**	**Patient health questionnaire-9**	***p*** **value**	**Burnout Measure-Short Version**	***p*** **value**
None	Median:9 (Min:3, Max:14)	0.01*	Median:4 (Min:1.7, Max:6)	0.005*
< 8 h	Median:9 (Min:0, Max:27)		Median:3.9 (Min:1, Max:7)	
8–16 h	Median:10 (Min:2, Max:23)		Median:4.2 (Min:1.3, Max:6.9)	
16–24 h	Median:11 (Min:0, Max:27)		Median:4.4 (Min:1, Max:7)	
24–32 h	Median:12 (Min:0, Max:27)		Median:4.5 (Min:1, Max:7)	
32–40 h	Median:10.5 (Min:0, Max:27)		Median:4.5 (Min:1,3, Max:7)	
> 40 h	Median:13 (Min:0, Max:27)		Median:4.5 (Min:1,4, Max:7)	
**COVID-19 positivity**	**Patient health questionnaire-9**	***p*** **value**	**Burnout Measure-Short Version**	***p*** **value**
Yes	Median:12 (Min:2, Max:27)	0.99**	Median:4.5 (Min:1.3, Max:7)	0.98**
No	Median:11 (Min:0, Max:27)		Median:4.3 (Min:1, Max:7)	
**Gender**^**Ω**^	**Patient health questionnaire-9**	***p*** **value**	**Burnout Measure-Short Version**	***p*** **value**
Female	Median:12 (Min:0, Max:27)	< 0.001**	Median:4.6 (Min:1.2, Max:7)	< 0,001*
Male	Median:10 (Min:0, Max:27)		Median:3.8 (Min:1, Max:7)	
**Type of residency**	**Patient health questionnaire-9**	***p*** **value**	**Burnout Measure-Short Version**	***p*** **value**
Full-time FM residents	Median:11 (Min:0, Max:27)	< 0.001**	Median:4.5 (Min:1, Max:7)	< 0.001**
CFMRs	Median:10 (Min:0, Max:27)		Median:3.95 (Min:1, Max:7)	
**Place of work**	**Patient health questionnaire-9**	***p*** **value**	**Burnout Measure-Short Version**	***p*** **value**
University Hospital	Median:11 (Min:0, Max:27)	0.003*	Median:4.3 (Min:1, Max:7)	< 0.001*
TRH	Median:12 (Min:0, Max:27)		Median:4.5 (Min:1.3, Max:7)	
PCC	Median:10 (Min:0, Max:27)		Median:4 (Min:1, Max:7)	
**PPE availability**	**Patient health questionnaire-9**	***p*** **value**	**Burnout Measure-Short Version**	***p*** **value**
All PPEs have been provided by the institution where they worked	Median:10,5 (Min:0, Max:27)	0.016*	Median:4.3 (Min:1.2, Max:7)	0.03*
Some PPEs have been provided by the institution where they worked	Median:11 (Min:0, Max:27)		Median:4.3 (Min:1, Max:7)	
Residents provided their own PPEs	Median:14 (Min:0, Max:27)		Median:5.3 (Min:1.3, Max:7)	

*Kruskal-Wallis test **Mann-Whitney U test (The calculation of the PHQ-9 and BMS were given in Table 1)

˟ Divorced and widowed participants were included in the Single group and evaluated

^Ω^ Since the number of participants who did not want to state their gender was insufficient, they were not included in the statistical analysis

As a result of Univariate Regression analysis, marital status, having children, average weekly contact time with COVID-19(+) patients, PPE availability, place of work and type of residency were found as explanatory factors for the PHQ-9 scale (Table 6). According to the Univariate Regression analysis, increase in the PHQ-9 scale were determined by the followings: 1.660 points for being single, 2.299 points for not having children, 0.576 points for contact with COVID-19(+) patients for more than 40 h per week, 2.977 points for the provision of PPEs by residents, 2.176 point for working in TRH, and 2.197 points for being a full-time FM resident. In the Multivariate Regression analysis model created with the variables found to be significant in the Univariate Regression analysis for the PHQ-9 scale, it was determined that the determinant factors were the average weekly contact time with COVID-19(+) patients (*p* < 0.01) and the PPE availability (*p* < 0.001).

**Table 6:** Univariate and Multivariate Regression analysis results with explanatory variables for PHQ-9

Patient health questionnaire-9	Univariate Regression Analysis				Multivariate Regression Analysis			
**Parameter**	B (std.err.)	95% CI		*p* value	B (std.err.)	95% CI		*p* value
**Marital status**	1.660 (0.598)	0.484	2.836	**0.006**	0.682 (0.672)	−0.639	2.002	0.311
**Having a child**	2.299 (0.632)	1.056	3.542	**< 0.001**	0.783 (0.817)	−0.822	2.389	0.338
**Gender**	3.729 (6.159)	−8.393	15.852	0.545	–	–	–	–
**Average weekly contact time with COVID-19(+) patients**	0.576 (0.156)	0.269	0.883	**< 0.001**	0.481 (0.175)	0.137	0.826	**0.006**
**PPE availability**	2.977 (0.911)	1.186	4.797	**0.001**	4.016 (0.924)	2.201	5.831	**< 0.001**
**Place of work**	2.176 (0.664)	0.872	3.479	**0.001**	1.275 (0.833)	−0.363	2.913	0.127
**Type of residency**	2.197 (0.655)	0.909	3.484	**0.001**	–	–	–	–

Type of residency was not included in the Multivariate Regression Model due to relating to the place of work

As a result of Univariate Regression analysis, marital status, having children, average weekly contact time with COVID-19(+) patients, PPE availability, place of work and type of residency were found as explanatory factors for the Burnout Measure-Short Version scale (Table 7). According to the Univariate Regression analysis, increase on the BMS scale were determined by the following: 0.416 points for being single, 0.519 points for not having children, 0.148 points for contact with COVID-19(+) patients for more than 40 h per week, 0.409 points for the provision of PPEs by residents, 0.652 points for working in TRH, and 0.654 points for being a full-time FM resident. In the Multivariate Regression analysis model created with the variables found to be significant in the Univariate Regression analysis for the BMS scale, it was determined that the determinant factors were the average weekly contact time with COVID-19(+) patients (*p* < 0.01), PPE availability (*p* < 0.001) and place of work (*p* < 0.01).

**Table 7:** Univariate and Multivariate Regression analysis results with explanatory variables for Burnout Measure-Short Version

Burnout Measure-Short Version	Univariate Regression Analysis				Multivariate Regression Analysis			
Parameter	B (std.err.)	95% CI		*p* value	B (std.err.)	95% CI		*p* value
**Marital status**	0.416 (0.131)	0.159	0.674	**0.002**	0.180 (0.147)	−0.109	0.469	0.222
**Having a child**	0.159 (0.139)	0.247	0.792	**0.000**	0.043 (0.173)	−0.297	0.384	0.803
**Gender**	1.099 (0.828)	−1.513	0.370	0.408	–	–	–	–
**Average weekly contact time with COVID-19(+) patients**	0.148 (0.034)	0.081	0.215	**0.000**	0.104 (0.038)	0.030	0.179	**0.006**
**PPE availability**	0.409 (0.200)	0.016	0.802	**0.041**	0.709 (0.200)	0.316	1.102	**0.000**
**Place of work**	0.652 (0.144)	00.370	0.935	**0.000**	0.477 (0.182)	0.119	0.834	**0.009**
**Type of residency**	0.654 (0.142)	0.375	0.093	**0.000**	–	–	–	–

Type of residency was not included in the Multivariate Regression Model due to relating to the place of work

## Discussion

The results of the present study show that, FM residents have spent less time in their departments, and instead, have taken on frontline duties in the fight against the COVID-19 pandemic. Both the duration of participation in educational activities and of working in their own departments decreased, resulting in less opportunity to engage with and learn FM core competencies and concepts.

We thought that all residents took an active role in the fight against COVID-19, regardless of seniority due to the insignificant difference between the time spent in residency and the average weekly contact time with COVID-19 positive patients.

The residents’ acquisition of clinical competencies outside the department was limited due to the reduction of both departmental training activities and rotations. 86.4% of residents stated that external rotations were negatively or very negatively affected (Table 4). The negative impact of the COVID-19 pandemic did not only affect FM residents, but also residents from other specialties. During the pandemic, residents were reported to work in most branches of the healthcare services and were assigned to departments outside their area of expertise, with consequent delay in specialty education [11, 25]. Suspended rotations, postponed face-to-face training and elective operations, due to the spread of COVID-19, were amongst the main problems that significantly affected resident training [25]. Residents, who may not be able to fulfill their clinical rotations, complete the minimum required surgeries and clinical competencies, may be subject to the greatest negative impact of this experience deficits [26]. In the United States (US), where the pandemic is intense, changes have been made in surgical residency programmes. 70% of the residents reported working in shifts, 31% reported suspension of some services, 33% reported limitation of annual leave, and 35% reported work re-assignment from non-surgical units to pandemic units [21].

On the basis of our study, CFMRs reported that no change was made to their residency training with a statistically significant higher frequency compared to other residents. It is thought that the probable cause for this situation is that CFMRs only work in the hospital during their rotation and go on such rotation for an average of only 3 months a year. The effect of the COVID-19 pandemic on their residencies could thus have been less than for full-time FM residents, as they continued to work in the PCC in the time period outside their rotation.

The present study shows that resident training hours decreased during the pandemic, with 90.2% of the residents stating that their training programmes were negatively or very negatively affected. In studies conducted with surgical residents in various places, most of the participants reported that COVID-19 had a negative effect on their clinical experience and training programmes [21, 27]. It has been recommended in various literature that resident training should be a priority even under pandemic conditions, and continuous education conferences could help residents to maintain a sense of ‘normalcy’ [15, 28]. The effects of preventive measures to minimize personal interactions during the COVID-19 pandemic is also reflected in training programmes. The effects of cancellations of face-to-face medical meetings were mitigated through recorded lectures, live broadcasts, and online seminars. There are even faculties that see the current pandemic conditions as an opportunity to begin mastering virtual learning [28]. Although webcasts have been increasingly adopted, face-to-face instructional lectures and experience transfer remain the cornerstone of medical education, and failure to sustain such traditional education methods negatively affected residents emotionally and spiritually [16, 29].

Almost 80% of the residents stated that their physical health was negatively or very negatively affected. Among surgical residents in the US, this rate has been reported as approximately 50% [21]. Less than one third of the institutions where residents trained provided all the necessary PPE, and this was associated with lower scores in the depression and burnout scales. In the literature, it has been observed that the lack of sufficient PPE can exacerbate burnout feelings amongst residents [30, 31]. PPE access is reported as protective not only in maintaining physical well-being, but also emotional and mental well-being [32, 33]. In a cross-sectional survey study conducted with general practitioners in Italy, it was reported that contact with COVID-19 positive patients with insufficient PPE was associated with increased depressive symptoms [32]. Mortagniti et al. conducted a study of 2707 health professionals from 60 countries, and reported that 51% of healthcare professionals suffered burnout during the pandemic [33]. Exposure to COVID-19 positive patients together with the consequent making of vital decisions increased burnout, whereas adequate and appropriate PPE was reported to be protective against burnout [33].

It should not be forgotten that working in healthcare is a source of stress in itself, even when there is no pandemic crisis [34, 35]. The fact that there are suicide attempts as well as exhaustion and physical stress in healthcare workers that result in death indicates mental stress [36]. The results of our survey showed that 90.6% of the residents stated that their mental health was negatively or very negatively affected during the COVID-19 pandemic. Previously, in healthcare workers taking care of COVID-19 patients, the prevalence of depression has been reported as 22.8–28% [37, 38]. According to the results of the PHQ-9 and BMS instruments, it was determined that while 30.6% of the residents suffered from moderately severe or severe depression, almost half had a very severe burnout problem or should seek professional help immediately. Depression and burnout levels amongst female residents were found to be higher in this study, similar to recent studies in the literature [21, 31]. In this study, it was noteworthy that married residents or those having children had lower levels of depression and burnout compared to single residents. This may be due to the improved social support for such doctors during the COVID-19 pandemic. According to published data from the US and Japan, depressive symptoms were also observed at higher rates in singles and women [39]. Difficult working conditions with increased stress, resource limitations, uncertain personal security, and increased patient morbidity and mortality during the COVID-19 pandemic have been compared to battlefield conditions. Healthcare providers caring for COVID-19 patients are known to have higher levels of burnout than those in other healthcare settings [40]. Such uncertainty and anxiety predispose residents to stress exposure syndromes such as post-traumatic stress disorder and burnout, as well as increased malpractice and inadequate patient care [12].

Almost 20% of the residents had been infected with COVID-19, and 4.2% of them stated that they continued to work without treatment. A previous study reported that 14.9% of COVID-19 positive residents working in New York City were not quarantined [41]. Given the expected future waves of the COVID-19 pandemic, it is thus suggested that appropriate strategies should be implemented to improve the safety of residents and the sustainability of clinical training [42, 43].

Almost 60% of the residents stated that their greatest concern relating to the pandemic was the fear of transmitting the virus to their families. In a study conducted in Saudi Arabia, 76.3% of residents and fellows were working in pandemic departments, and more than half of the doctors stated that they were mostly concerned about the safety of their families [16]. As with healthcare workers previously, residents were trying to manage their physical and mental health integrity, while having to balance the needs of patients with their own needs and those of their families [44].

The exposure of residents to COVID-19 positive patients is associated with increased burnout [45]. In the present study, it was observed that the duration of contact with COVID-19 positive patients affected residents’ depression and burnout scale scores. Likewise, it was observed that residents working in TRH and University Hospitals, who were frequently assigned to pandemic outpatient clinics and wards, were more likely to have had COVID-19 infection when compared to residents working in PCC. This study determined that COVID-19 infection in residents correlated with serious burnout problems. It has been reported in the literature that doctors who come into direct contact with suspected or confirmed cases of COVID-19 in hospitals are the healthcare professionals most susceptible to this disease, and this situation has multiple negative effects on mental health [46, 47].

Although the sequelae of this pandemic crisis on residents cannot be determined until pandemic eradication, the results of this study reveal what residents have been experiencing throughout the COVID-19 pandemic. It has been put forward that organizational and social psychological counselling and support will reduce mental health problems in healthcare workers during the pandemic [48, 49]. In addition, the support needed by frontline health workers should include basic needs, PPE availability, and childcare support. Counselling sessions and the availability of anonymous psychological support resources (e.g. crisis hotlines), and ensuring the continuation of existing support are considered crucial for health of healthcare workers [50, 51]. We want to emphasize that as healthcare professionals, we need to think about what we can do not only for our patients, but also for our colleagues, juniors, residents and ourselves, for our physical and mental well-being.

Although this is the first study of the effect of the COVID-19 pandemic on FM residents in Turkey, it has the usual limitations of self-administered surveys, especially considering the low response rate. The questionnaire aims to determine FM residents’ perceptions of the impact of the COVID-19 pandemic on their mental health and residency training. Since the participation was based on a voluntary basis, it is possible that residents who have more interest in the subject and have more heightened negative experiences may participate more, resulting in a possible volunteer bias. In addition, the seniority of the residents and their place of work at the time of answering the questionnaire may have influenced their perceptions. The exclusion of incomplete questionnaires from the analysis was another limitation of the present study. It is possible that incomplete questionnaires and omission of answers may have led to insights not previously considered. Another possible limitation, as this study was conducted during the pandemic period, is that it may not reflect the chronic consequences of stress associated with the pandemic. Long-term prospective studies may better reflect the cause-and-effect relationship between the pandemic conditions and residents’ physical and mental health. However, the detection of important findings including the depression and burnout states of residents, lack of training opportunities, and the requirement of necessary precautions reveal the importance of the study.

## Conclusions

As in other worldwide disciplines [16, 48, 52], the COVID-19 pandemic has had an adverse impact on FM training programmes for residents working in the frontline during the COVID-19 pandemic in Turkey. This creates the need for educational institutions to create a consensus to develop and implement innovative education techniques to compensate for such deficiencies. Heavy workload and disruption in residency training caused by the pandemic is closely related to increased depression and burnout. Due to the unpredictability of the pandemic, evidence-based decisions, and long-term action plans should be implemented to better address the educational needs of residents, as learners and frontline care providers, and to protect their physical and mental health.

## Supplementary Information

**Additional file 1.**

## Data Availability

All data generated or analyzed during this study are included in this article.

## Abbreviations

PHQ-9: Patient Health Questionnaire-9
BMS: Burnout Measure-Short Version
WHO: World Health Organization
NCD: Non-communicable diseases
PCC: Primary care clinics
FM: Family medicine
FMS: FM Specialists
PPE: Personal protective equipment
CFMR: Contracted FM residents
TRH: Training and Research Hospital
US: United States

## References

[1] Jebril N. World Health Organization declared a pandemic public health menace: A systematic review of the coronavirus disease 2019 “COVID-19”, up to 26th March 2020. Available at SSRN 3566298 2020.

[2] Genç K (2021). COVID-19 in Turkey: a nation on edge. Lancet.

[3] T.C. Sağlık Bakanlığı COVID-19 Bilgilendirme Platformu [https://covid19.saglik.gov.tr/].

[4] COVID-19: Turkey announces full lockdown from Thursday [https://www.aa.com.tr/en/latest-on-coronavirus-outbreak/covid-19-turkey-announces-full-lockdown-from-thursday-/2221503].

[5] Şimşek AÇ, Kara A, Baran-Aksakal FN, Gülüm M, Ilter B, Ender L, Bulut YE, Gül H, Irmak H, Altunay K. Contact tracing management of the COVID-19 pandemic. Türk Hijyen Deneysel Biyoloji Dergisi. 2020;77(3):269–80.

[6] Panocchia N, D'Ambrosio V, Corti S, Lo Presti E, Bertelli M, Scattoni ML, Ghelma F. COVID-19 pandemic, the scarcity of medical resources, community-centred medicine and discrimination against persons with disabilities. J Med Ethics. 2021;47:362–6. 10.1136/medethics-2020-107198 33827907

[7] Sahin B, Hanalioglu S. The Continuing Impact of Coronavirus Disease 2019 on Neurosurgical Training at the 1-Year Mark: Results of a Nationwide Survey of Neurosurgery Residents in Turkey. World Neurosurg. 2021;151:e857–e870. 10.1016/j.wneu.2021.04.137 PMC9760305 33974985

[8] Caruana EJ, Patel A, Kendall S, Rathinam S (2020). Impact of coronavirus 2019 (COVID-19) on training and well-being in subspecialty surgery: a national survey of cardiothoracic trainees in the United Kingdom. J Thorac Cardiovasc Surg.

[9] World Health Organization (2020). Role of primary care in the COVID-19 response.

[10] Ardıç C. COVID-19 Pandemi Süreci. *Türkiye Klinikleri* 2020, 1. Baskı(Aile Hekimliği ve COVID-19 Pandemisi):5–7.

[11] Nasrallah MS, Tawfik HA, Aseel MT (2020). Family medicine residency training program during COVID19: Qatari experience. Pan Afr Med J.

[12] Preti E, Di Mattei V, Perego G, Ferrari F, Mazzetti M, Taranto P, Di Pierro R, Madeddu F, Calati R (2020). The psychological impact of epidemic and pandemic outbreaks on healthcare workers: rapid review of the evidence. Curr Psychiatry Rep.

[13] Sharif S, Amin F, Hafiz M, Benzel E, Peev N, Dahlan RH, Enchev Y, Pereira P, Vaishya S, Board WSCSE (2020). COVID 19–depression and neurosurgeons. World Neurosurg.

[14] Magnavita N, Chirico F, Garbarino S, Bragazzi NL, Santacroce E, Zaffina S. SARS/MERS/SARS-CoV-2 outbreaks and burnout syndrome among healthcare workers. An umbrella systematic review. Int J Environ Res Public Health. 2021;18(8):1–13. 10.3390/ijerph18084361 PMC8072681 33924026

[15] Rakowsky S, Flashner BM, Doolin J, Reese Z, Shpilsky J, Yang S, Smith CC, Graham K. Five questions for residency leadership in the time of COVID-19: reflections of chief medical residents from an internal medicine program. Acad Med. 2020;95(8):1152–4. 10.1097/ACM.0000000000003419 PMC7179057 32287083

[16] Balhareth A, AlDuhileb MA, Aldulaijan FA, Aldossary MY (2020). Impact of COVID-19 pandemic on residency and fellowship training programs in Saudi Arabia: a nationwide cross-sectional study. Ann Med Surg.

[17] Dimitriu MCT, Pantea-Stoian A, Smaranda AC, Nica AA, Carap AC, Constantin VD, Davitoiu AM, Cirstoveanu C, Bacalbasa N, Bratu OG (2020). Burnout syndrome in Romanian medical residents in time of the COVID-19 pandemic. Med Hypotheses.

[18] Sari YE, Kokoglu B, Balcioglu H, Bilge U, Colak E, Unluoglu I (2016). Turkish reliability of the patient health questionnaire-9. Biomed Res India.

[19] Capri B (2013). The Turkish adaptation of the burnout measure-short version (BMS) and couple burnout measure-short version (CBMS) and the relationship between career and couple burnout based on psychoanalytic-existential perspective. Educ Sci Theory Pract.

[20] Chang DG, Park JB, Baek GH, Kim HJ, Bosco A, Hey HWD, Lee CK. The impact of COVID-19 pandemic on orthopaedic resident education: a nationwide survey study in South Korea. Int Orthop. 2020;44(11):2203–10. 10.1007/s00264-020-04714-7 PMC7351541 32651712

[21] Coleman JR, Abdelsattar JM, Glocker RJ. COVID-19 Pandemic and the lived experience of surgical residents, fellows, and early-career surgeons in the American College of Surgeons. J Am Coll Surg. 2021;232(2):119–135.e120. 10.1016/j.jamcollsurg.2020.09.026 PMC7561602 33069850

[22] Kroenke K, Spitzer RL, Williams JB (2001). The PHQ-9: validity of a brief depression severity measure. J Gen Intern Med.

[23] Malach-Pines A (2005). The burnout measure, short version. Int J Stress Manag.

[24] World Health Organization: WHO COVID-19: Case definitions [WHO/2019-nCoV/Surveillance_Case_Definition/2020.1]. Geneva: World Health Organization; 2020.

[25] McCarthy C, Carayannopoulos K, Walton JM (2020). COVID-19 and changes to postgraduate medical education in Canada. CMAJ.

[26] Potts JR (2020). Residency and fellowship program accreditation: effects of the novel coronavirus (COVID-19) pandemic. J Am Coll Surg.

[27] Pertile D, Gallo G, Barra F, Pasculli A, Batistotti P, Sparavigna M, Vizzielli G, Soriero D, Graziano G, Di Saverio S (2020). The impact of COVID-19 pandemic on surgical residency programmes in Italy: a nationwide analysis on behalf of the Italian Polyspecialistic young surgeons society (SPIGC). Updat Surg.

[28] Ehrlich H, McKenney M, Elkbuli A (2020). We asked the experts: virtual learning in surgical education during the COVID-19 pandemic-shaping the future of surgical education and training. World J Surg.

[29] Singh K, Srivastav S, Bhardwaj A, Dixit A, Misra S (2020). Medical education during the COVID-19 pandemic: a single institution experience. Indian Pediatr.

[30] Dedeilia A, Sotiropoulos MG, Hanrahan JG, Janga D, Dedeilias P, Sideris M (2020). Medical and surgical education challenges and innovations in the COVID-19 era: a systematic review. In Vivo.

[31] Alrawashdeh HM, Al-Tammemi AB, Alzawahreh MK, Al-Tamimi A, Elkholy M, Al Sarireh F, Abusamak M, Elehamer NMK, Malkawi A, Al-Dolat W (2021). Occupational burnout and job satisfaction among physicians in times of COVID-19 crisis: a convergent parallel mixed-method study. BMC Public Health.

[32] Amerio A, Bianchi D, Santi F, Costantini L, Odone A, Signorelli C, Costanza A, Serafini G, Amore M, Aguglia A (2020). Covid-19 pandemic impact on mental health: a web-based cross-sectional survey on a sample of Italian general practitioners. Acta Biomed Atenei Parmensis.

[33] Morgantini LA, Naha U, Wang H, Francavilla S, Acar Ö, Flores JM, Crivellaro S, Moreira D, Abern M. Eklund M, et al. Factors Contributing to Healthcare Professional Burnout During the COVID-19 Pandemic: A Rapid Turnaround Global Survey. PLOS ONE. 2020;15(9):e0238217. 10.1371/journal.pone.0238217 PMC7470306 32881887

[34] Magnavita N, Garbarino S (2013). Social psychiatry in the waiting room: what a physician can learn about occupational stress from workers waiting to be examined. Psychiatry J.

[35] Magnavita N, Fileni A (2014). Association of work-related stress with depression and anxiety in radiologists. Radiol Med.

[36] Ing EB, Xu QA, Salimi A, Torun N (2020). Physician deaths from corona virus (COVID-19) disease. Occup Med (Lond).

[37] Luo M, Guo L, Yu M, Jiang W, Wang H (2020). The psychological and mental impact of coronavirus disease 2019 (COVID-19) on medical staff and general public - a systematic review and meta-analysis. Psychiatry Res.

[38] Pappa S, Ntella V, Giannakas T, Giannakoulis VG, Papoutsi E, Katsaounou P (2020). Prevalence of depression, anxiety, and insomnia among healthcare workers during the COVID-19 pandemic: a systematic review and meta-analysis. Brain Behav Immun.

[39] Inaba A, Thoits PA, Ueno K, Gove WR, Evenson RJ, Sloan M (2005). Depression in the United States and Japan: gender, marital status, and SES patterns. Soc Sci Med.

[40] Ruiz-Fernández MD, Ramos-Pichardo JD, Ibáñez-Masero O, Cabrera-Troya J, Carmona-Rega MI, Ortega-Galán ÁM (2020). Compassion fatigue, burnout, compassion satisfaction and perceived stress in healthcare professionals during the COVID-19 health crisis in Spain. J Clin Nurs.

[41] Breazzano MP, Shen J, Abdelhakim AH, Glass LRD, Horowitz JD, Xie SX, de Moraes CG, Chen-Plotkin A, Chen RWS, on behalf of the New York City Residency Program Directors COVID-19 Research Group. New York City COVID-19 resident physician exposure during exponential phase of pandemic. J Clin Invest. 2020;130(9):4726–33. 10.1172/JCI139587 PMC7456242 32463802

[42] Anton M, Wright J, Braithwaite M, Sturgeon G, Locke B, Milne C, Crosby A. Creating a COVID-19 Action Plan for GME Programs. J Grad Med Educ. 2020;12(4):399–402. 10.4300/JGME-D-20-00206.1 PMC7450757 32879675

[43] Leung K, Wu JT, Liu D, Leung GM. First-wave COVID-19 transmissibility and severity in China outside Hubei after control measures, and second-wave scenario planning: a modelling impact assessment. Lancet. 2020;395(10233):1382–93. 10.1016/S0140-6736(20)30746-7 PMC7195331 32277878

[44] Greenberg N, Docherty M, Gnanapragasam S, Wessely S (2020). Managing mental health challenges faced by healthcare workers during covid-19 pandemic. Bmj.

[45] Kannampallil TG, Goss CW, Evanoff BA, Strickland JR, McAlister RP, Duncan J (2020). Exposure to COVID-19 patients increases physician trainee stress and burnout. PLoS One.

[46] Lima CKT, Carvalho PMM, Lima I, Nunes J, Saraiva JS, de Souza RI, da Silva CGL, Neto MLR. The emotional impact of Coronavirus 2019-nCoV (new Coronavirus disease). Psychiatry Res. 2020;287:112915. 10.1016/j.psychres.2020.112915 PMC7195292 32199182

[47] Xiang Y-T, Yang Y, Li W, Zhang L, Zhang Q, Cheung T, Ng CH (2020). Timely mental health care for the 2019 novel coronavirus outbreak is urgently needed. Lancet Psychiatry.

[48] Alshdaifat E, Sindiani A, Khasawneh W, Abu-Azzam O, Qarqash A, Abushukair H, Obeidat N (2021). The impact of COVID-19 pandemic on training and mental health of residents: a cross-sectional study. BMC Med Educ.

[49] De Brier N, Stroobants S, Vandekerckhove P, De Buck E (2020). Factors affecting mental health of health care workers during coronavirus disease outbreaks (SARS, MERS & COVID-19): a rapid systematic review. PLoS One.

[50] Lee TH, Zhang C, Wood R, Kothari D (2021). C.O.V.I.D.: a survival guide for GI fellowship training during the COVID-19 pandemic. Clin Gastroenterol Hepatol.

[51] Dzau VJ, Kirch D, Nasca T (2020). Preventing a parallel pandemic - a National Strategy to protect Clinicians' well-being. N Engl J Med.

[52] Anwar A, Seger C, Tollefson A, Diachun CAB, Tanaka P, Umar S (2020). Medical education in the COVID-19 era: impact on anesthesiology trainees. J Clin Anesth.

