# Supplementary material for: The impacts of the COVID-19 pandemic on the mental health and residency training of family medicine residents: findings from a nationwide cross-sectional survey in Turkey
Source: BMC Fam Pract. 2021 Nov 15;22:226. doi: 10.1186/s12875-021-01576-9 (PMC8591155; doi:10.1186/s12875-021-01576-9)
Supplement: Supplementary file 1 — Additional file 1. [file 12875_2021_1576_MOESM1_ESM.docx]

**QUESTIONNAIRE**

***Section 1: Sociodemographic Information***

**Age** ………

**Gender** ( ) Female ( ) Male ( ) Don’t want to specify

**Marital status**

( ) Married

( ) Single

( ) Divorced

( ) Widowed

**Do you have children?**

( ) Yes

( ) No

***Section 2: Details of the Residency Program***

**Duration of medical practice** ..... (years)

**Time spent in Family Medicine residency** …… (months)

**Place of work**

( ) University Hospital

( ) Training and Research Hospital

( ) Primary Care Clinic

**Type of residency**

( ) Full-time Family Medicine resident

( ) Contracted Family Medicine resident

**Average working time per week (hours)**

|  | Before the COVID-19 Pandemic | During the COVID-19 Pandemic |
| --- | --- | --- |
| <24 hours |  |  |
| 24-40 hours |  |  |
| 40-56 hours |  |  |
| 56-72 hours |  |  |
| >72 hours |  |  |

**Average working time per week in the FM clinic (hours)**

|  | Before the COVID-19 Pandemic | During the COVID-19 Pandemic |
| --- | --- | --- |
| <8 hours |  |  |
| 8-16 hours |  |  |
| 16-24 hours |  |  |
| 24-32 hours |  |  |
| >32 hours |  |  |

**Duration of participation in training programs per week (hours)**

|  | Before the COVID-19 Pandemic | During the COVID-19 Pandemic |
| --- | --- | --- |
| <1 hour |  |  |
| 1-3 hours |  |  |
| 3-5 hours |  |  |
| >5 hours |  |  |

**Please mark the education programs you attended before and during the COVID-19 pandemic. (Tick all appropriate options)**

|  | Face to face before the COVID-19 pandemic | Online before the COVID-19 pandemic | Face to face during the COVID-19 pandemic | Online during the COVID-19 pandemic |
| --- | --- | --- | --- | --- |
| Literature research |  |  |  |  |
| Seminars and/or lectures delivered by residents |  |  |  |  |
| Seminars and/or lectures delivered by faculty members at the institution |  |  |  |  |
| Seminars and/or lectures delivered by faculty members from another institution |  |  |  |  |

**Which changes have been made in the residency program due to the COVID-19 pandemic? (Tick all appropriate options)**

( ) No changes were made.

( ) Residents were dismissed from some services.

( ) Residents were working shifts.

( ) Unable to obtain leave from work (although the Ministry of Health had officially stated that annual leave could be taken)

( ) Able to take leave after official notification

( ) Residents were assigned to outpatient clinics and services other than FM

( ) Some changes had been made temporarily, but the residency program had then returned to normal.

**How has the COVID-19 pandemic impacted your expertise in the following areas?**

|  | **Very negatively affected** | **Negatively affected** | **Not affected** | **Positively affected** | **Very positively affected** |
| --- | --- | --- | --- | --- | --- |
| Education programs |  |  |  |  |  |
| Educational experience in inpatient clinics |  |  |  |  |  |
| Educational experience in outside rotations |  |  |  |  |  |
| Physical health |  |  |  |  |  |
| Mental health |  |  |  |  |  |

**Do you think that the specialists and/or faculty members in your clinic take the same level of risk compared to the residents?**

( ) No, specialist and/or faculty members took less risk.

( ) Yes, the same level of risk

( ) No, specialist and/or faculty members took higher levels of risk.

**Do you feel that assistants are treated equally compared to specialists and/or faculty members during the COVID-19 pandemic in your institution?**

( ) Yes, treated equally.

( ) No, there was inequality.

***Section 3: Contact with COVID-19 patients***

**Average weekly contact time with COVID-19 (+) patients (hours)**

| ( ) None  ( ) <8 hours |
| --- |
| ( ) 8-16 hours |
| ( ) 16-24 hours |
| ( ) 24-32 hours |
| ( ) 32-40 hours  ( ) >40 hours |

**Location of contact with patients with suspected or confirmed COVID-19**

( ) FM outpatient clinics

( ) COVID-19 outpatient clinic

( ) Emergency Department

( ) COVID-19 inpatient treatment unit

( ) COVID-19 Intentive Care Unit (ICU)

( ) Contact tracing (Filiation studies)

***Section 4:* What is your *greatest concern during the COVID-19 pandemic?***

( ) Clinical disability

( ) Falling behind in education

( ) Ethical issues

( ) The fear of catching COVID-19

( ) The fear of carrying COVID-19 to the family

***Section 5: Protection from COVID-19***

**Did you receive ~~a~~ specific training on how to deal with COVID-19 and use PPE?**

( ) I received training before the first case was reported in Turkey.

( ) I received training after the first case was reported in Turkey.

( ) I have not receive training at any time.

**PPE availability**

( ) All PPEs are being provided by the institution where I work

( ) I have been providing my own PPE

( ) Some ppes are beıng provided by the institution where I work

**Have you being infected with COVID-19?**

( ) Yes

( ) No

**Treatment and follow-up (Only to be answered by the participants who have been infected with COVID-19.)**

( ) Continued working without treatment

( ) Stayed in quarantine without receiving treatment

( ) Treated at home

( ) Received inpatient treatment

( ) Received treatment in ICU

***Section 6: Mental Health***

**Patient Health Questionnaire-9**

| How often have they been bothered by the following over the past 2 weeks? | Not at all | Several days | More than half the days | Nearly every day |
| --- | --- | --- | --- | --- |
| Little interest or pleasure in doing things? | 0 | 1 | 2 | 3 |
| Feeling down, depressed, or hopeless? | 0 | 1 | 2 | 3 |
| Trouble falling or staying asleep, or sleeping too much? | 0 | 1 | 2 | 3 |
| Feeling tired or having little energy? | 0 | 1 | 2 | 3 |
| Poor appetite or overeating? | 0 | 1 | 2 | 3 |
| Feeling bad about yourself - or that you are a failure or have let yourself or your family down? | 0 | 1 | 2 | 3 |
| Trouble concentrating on things, such as reading the newspaper or watching television? | 0 | 1 | 2 | 3 |
| Moving or speaking so slowly that other people could have noticed? Or so fidgety or restless that you have been moving a lot more than usual? | 0 | 1 | 2 | 3 |
| Thoughts that you would be better off dead, or thoughts of hurting yourself in some way? | 0 | 1 | 2 | 3 |

**The Burnout Measure – Short Version**

Please use the following scale to answer the question: When you think about your work overall, how often do you feel the following?

|  | Never | Almost never | Rarely | Sometimes | Often | Very often | Always |
| --- | --- | --- | --- | --- | --- | --- | --- |
| Tired | 1 | 2 | 3 | 4 | 5 | 6 | 7 |
| Disappointed with people | 1 | 2 | 3 | 4 | 5 | 6 | 7 |
| Hopeless | 1 | 2 | 3 | 4 | 5 | 6 | 7 |
| Trapped | 1 | 2 | 3 | 4 | 5 | 6 | 7 |
| Helpless | 1 | 2 | 3 | 4 | 5 | 6 | 7 |
| Depressed | 1 | 2 | 3 | 4 | 5 | 6 | 7 |
| Physically weak/Sickly | 1 | 2 | 3 | 4 | 5 | 6 | 7 |
| Worthless/Like a failure | 1 | 2 | 3 | 4 | 5 | 6 | 7 |
| Difficulties sleeping | 1 | 2 | 3 | 4 | 5 | 6 | 7 |
| “I’ve had it.” | 1 | 2 | 3 | 4 | 5 | 6 | 7 |
